# The challenges characterizing the lived experience of caregiving. A qualitative study in the field of spinal cord injury

**DOI:** 10.1038/s41393-021-00618-4

**Published:** 2021-03-19

**Authors:** Claudia Zanini, Julia Amann, Mirjam Brach, Armin Gemperli, Sara Rubinelli

**Affiliations:** 1 Swiss Paraplegic Research, Nottwil, Switzerland; 2 Department of Health Sciences and Medicine, University of Lucerne, Lucerne, Switzerland; 3 Department of Health Sciences and Technology, Health Ethics and Policy Lab, ETH, Zurich, Switzerland

**Keywords:** Quality of life, Risk factors

## Abstract

**Study design:**

Qualitative exploratory study.

**Objectives:**

To explore the lived experience of SCI caregivers, with a focus on the challenges of their role.

**Setting:**

Caregivers of people with SCI living in the community in Switzerland.

**Methods:**

Data were collected through semi-structured interviews. Thematic analysis was performed.

**Results:**

The sample included 22 participants (16 women, 15 life partners) with a mean age of 61 years who had been caregivers for an average of 18 years. Caregiving in SCI seemed to be characterized by two phases. The first phase was relatively short and was central to becoming a caregiver; it was marked by challenges related to adjusting to the role of caregiver (e.g., dealing with shock, feeling unprepared). The second phase is lifelong and is characterized by a number of recurrent challenges related to balancing caregiving and personal life (e.g., having to prioritize caregiving over personal wishes, negotiating tasks and workload). Challenges related to lacking appropriate housing, facing financial uncertainty and dealing with bureaucracy were noted during both phases. Caregivers had to deal with these challenges to stay in step with life changes and newly emerging needs.

**Conclusions:**

Informal caregivers have a major role in supporting people with SCI. But their needs are not static. Any strategy to empower them has to adapt to an evolving role characterized by multiple tasks and challenges. A functional relationship between caregivers and care recipients is based on the recognition of their individualities and the different phases of adaptation, which is also an enriching process.

## Introduction

A spinal cord injury (SCI) changes a person’s life overnight, requiring them to relearn the most basic tasks. People with a SCI who do not achieve complete independence rely on support to manage their condition and carry out daily activities. This support contributes to their quality of life and ability to participate in society and is often provided by family members [1]. Moreover, caregivers provide support not only for mobility and household chores, but also for respiratory care, body care, bowel and bladder management, and eating and drinking [2, 3]

Research in the field of other chronic conditions, such as cancer, Alzheimer’s disease, and Parkinson’s disease, shows that caregiving often disrupts the life of the caregiver, including their professional, social, and familial relationships [1, 4–6]. Furthermore, studies have reported the perceived impact that the role of caregiving has on a caregiver’s life (e.g., caregiver burden) [7, 8].

In the context of SCI, recent reviews of the literature have reported caregivers having high levels of burden, depression and anxiety, physical symptoms, and reduced life satisfaction, as well as feelings of isolation and a loss of identity [9, 10]. This might be due to the fact that, unlike caregiving in other conditions, caregiving in SCI can last decades, as the life expectancy of people with SCI has increased, and these caregivers often take on the role in early-middle age [9, 11].

Caregiving is an evolving experience that is shaped by an interplay of contextual and relational factors. To better support caregivers, it is necessary to identify the challenges they encounter in caring for a loved one and assess the appropriateness of the healthcare system’s response to their needs [12]. This study will explore the lived experience of SCI caregivers, with a focus on the challenges that they face over time. While research in the field of SCI has emphasized the caregiver burden (e.g., predictors, impact on health) [3, 13, 14], this study looks at the evolution of the role of caregiver as experienced by those in this position.

## Methods

### Study design, sampling, and recruitment

This qualitative exploratory study is part of a larger project about informal caregivers of people with SCI in Switzerland. The project included a quantitative study [2] and a qualitative study with a subgroup of participants. Overall, the latter aimed to identify targets for the empowerment of informal caregivers in terms of support, information services and self‐management resources. Some of its findings are presented in this manuscript.

To recruit participants, we contacted the people with SCI included in the address lists of the national longitudinal survey (Swiss Spinal Cord Injury Cohort Study) [15, 16], cross-referenced with the membership database of the Swiss Paraplegic Association and patient databases of the four SCI-specialized clinics in Switzerland. Those with SCI were asked to give the enclosed survey to their primary family caregiver. The caregivers who completed the survey (*N* = 717) could indicate in the informed consent form their availability to take part in an interview.

All the caregivers who indicated in the survey, their availability to take part in an interview, were considered potential participants. Among them, we recruited a purposive sample of participants for this study. The participants fulfilled the following inclusion criteria: being >18 years of age, speaking an official Swiss language (French, German, or Italian) fluently, providing at least 10 h per week of care to a family member with SCI who has been a wheelchair user for at least 4 years, and perceiving either a high or low burden in relation to caregiving. This information was retrieved from the survey. To capture a wide range of perspectives on SCI caregiving, we also considered factors that might affect the experience of caregiving [17–20] such as gender and age (for caregiver and care recipient), linguistic region, relationship between caregiver and care recipient (partner, parent, adult child), financial expenditures due to caregiving, and number of years of caregiving.

The potential participants were contacted via phone or via email, depending on the contact method that they indicated as preferred in the survey. We tried to join 34 caregivers, of which 22 actually participated in the study. We stopped trying to recruit a potential participant after having called three times at different times of the day or after having sent two reminders without answer.

Participation in the study was voluntary, and written consent was obtained from all interviewees. During the first contact, the researcher would quickly present the aim of the study and what the participation consisted of. If the person showed an interest in participating, the study information and a copy of the informed consent were sent home. Later on, the interview was scheduled. The day of the interview, the researcher would remind the most important issues linked to the declaration of informed consent and the interviewee would sign the document.

### Data collection

Data were collected through audio-recorded semi-structured interviews. The interview guide was developed by CZ and revised in collaboration with the research team. The questions explored the experiences of caregiving and their evolution over time. Knowing from the literature that caregiving can be a disruptive event and that caregivers often reported negative outcomes, we included questions that covered these aspects. In addition, and in line with other scientific literature, we asked questions to uncover the positive aspects of caregiving, as well as resources and strategies that caregivers put into place. The main questions of the interview guide are presented in Table 1. The questions would guide the conversation but there was the opportunity to follow-up on issues raised by the participants.

**Table 1:** Sample questions from the interview grid.

Topics	Leading questions	Additional questions
Freedom of choice	What factors influenced your decision to care for [CR]?	• Was professional care considered?
Skills	Which are in your opinion the most important skills a caregiver in your situation would need?	
Role/self-efficacy	How do you feel in your role of caregiver?	• Do you feel confident? At ease? Why? • What helps you deal well with this activity?
Impact of caregiving on personal life	How has your life changed since you took over caregiving?	• Which aspects of your life have been affected by this activity? • What was mainly affected?
Positive aspects of caregiving	What are the positive aspects of being a caregiver?	
Negative aspects of caregiving	What do you think are difficult aspects of being a caregiver? Why?	• Difficult tasks • Organization private and professional life with the caregiving • Time for yourself
Coping strategies	How are you coping with the responsibilities of caregiving?	• How do you deal with practical problems related to caregiving that you encounter in daily life?
General support	Overall, how do you feel supported in your role of caregiver?	• From whom? • Which kind of support? • What helps you most? Why?
Professional support	What about professional support for yourself?	• Are you aware of the possibilities for support? • Are you using any of them?
Needs and suggestions	Is there anything that could help you better deal with your caregiving tasks?	• Information • Wishes?
Needs and suggestions	How do you feel when looking at the future?	• Fear or worry for yourself/the CR?

Three pilot interviews were conducted to test the interview guide after which no major changes were made, but the wording was improved. The pilot interviews were therefore included in the analysis.

The first author and a trained research assistant (NL) conducted all interviews in the interviewees’ preferred language (German, French, or Italian) and in a location of their choosing (i.e., home, workplace, bar). The interviewers kept a field diary in which they took notes of initial analysis thoughts, interpretations, and questions as well as of feelings during the interviews and first impressions. The interviewers also discussed issues emerged in the conduction of the interviews (e.g., how to best support a caregiver who is crying, how to deal with the own emotions).

### Data analysis

The interviews lasted an average of 70 min (±SD = 33), were transcribed verbatim and analyzed following the principles of thematic analysis [21]. The analysis included both a deductive and an inductive phase: The first author (CZ) conducted the first deductive coding of six interviews and JA and NL of each three interviews. The three researchers met then to compare the coding and solve disagreement. A similar procedure was followed for the inductive coding. The data of each theme were constantly compared to ensure their homogeneity as well as their distinctiveness from other themes. Thematic saturation was reached.

To ensure trustworthiness, the researchers involved in the analysis documented their personal reflections on the data and had regular informal peer debriefings. They also performed investigator triangulation (e.g., by checking preliminary findings and interpretations against the raw data) to reduce researcher bias. They kept track of their discussions on themes, labeling, etc. to remember how and why decisions were made.

The interviews were analyzed in their original language, and excerpts were translated only for the purpose of scientific publications. The software MAXQDA™ (Release 12.2.0) was used to organize and store the data.

More details are presented in the Supplementary file 1.

## Results

The final sample included 22 participants (16 women) with a mean age of 61 years (±SD = 10.4) who had been caregivers for an average of 18 years (±SD = 13.5). The majority of the participants were the life partners (*n* = 15) of the care recipient. Participants’ characteristics are presented in Table 2.

**Table 2:** Participants’ characteristics.

Interview ID	Age	Years of caregiving	Gender caregiver	Relationship to the care recipient	Care recipient’s SCI type
INT01	73	6	F	Life partner–together before SCI onset	Paraplegia
INT02	76	10	M	Life partner–together before SCI onset	Paraplegia
INT03	64	22	F	Parent	Paraplegia
INT04	65	11	M	Life partner–together before SCI onset	Paraplegia
INT05	53	42	F	Adult child	Paraplegia
INT06	58	7	F	Parent	Tetraplegia
INT07	70	5	M	Life partner–together before SCI onset	Tetraplegia
INT08	50	16	F	Life partner–together before SCI onset	Tetraplegia
INT09	48	23	M	Life partner–got to know after SCI onset	Tetraplegia
INT10	66	39	F	Life partner–together before SCI onset	Tetraplegia
INT11	72	27	F	Parent	Tetraplegia
INT12	35	13	M	Life partner–together before SCI onset	Tetraplegia
INT13	47	11	F	Life partner–together before SCI onset	Paraplegia
INT14	57	5	F	Parent	Paraplegia
INT15	77	5	M	Life partner–together before SCI onset	Paraplegia
INT16	63	17	F	Life partner–got to know after SCI onset	Paraplegia
INT17	69	47	F	Life partner–together before SCI onset	Paraplegia
INT18	57	6	F	Parent	Tetraplegia
INT19	63	19	F	Sibling	Tetraplegia
INT20	60	41	F	Life partner–together before SCI onset	Tetraplegia
INT21	54	24	F	Life partner–together before SCI onset	Tetraplegia
INT22	59	5	F	Life partner–together before SCI onset	Tetraplegia

The analysis indicated that the experience of SCI caregiving had two phases. The first phase covered the period in which the family member became a caregiver. In most cases, this happened after the patient’s discharge from their first rehabilitation. However, in some cases, caregiving began later (e.g., partners met after SCI onset and became caregivers once they moved in together). The second phase began after a new routine had been arranged.

Many participants claimed the beginning of caregiving was a “natural” consequence of the relationship they had with the care recipient, anchored in social norms (e.g., a wife is expected to take care of her husband) and an expectation of reciprocity (i.e., she/he would have done it for me) (illustrative quotes are presented in Table 3, Q1). In their views, being a caregiver was a part of their duties as a mother or life partner (Q2) and some explained that helping was part of their nature or personality (Q3). Among the reasons mentioned for having become caregivers, the participants often referred to a desire to protect the family privacy and to avoid dependence on homecare providers (Q4). However, in other cases, it was the concurrence of certain life circumstances (e.g., being unemployed and a nurse by training when the accident happened); expressions like “I slipped into it” stressed the accidental way the caregiving began (Q5). Other reasons for “slipping into caregiving” were not finding any appropriate assisted living facility or the expectation of the injured person to be assisted by the family member (Q6).

**Table 3:** Illustrative quotes.

Q1	• We are still the old married couple who say yes to the end [laughs]. It goes without saying [that I took on caregiving], my husband would have done the same for me, there was not even a discussion (INT01) • We wanted to be together from the very beginning and I probably would have had similar expectations, although she said things like, she didn’t take it for granted that I’m still here now. […] I never really had to think about that. That’s why I got married, “be together for better and for worse”. I think this statement fits well. (INT21) • For me it was never a question, “Am I going to do this?” That was very clear. It’s simple. I love my child and never in your life is a person as close as your child is. (INT18) • The upbringing. You look after each other. At the beginning of life, the child is dependent on the mother and at some point it turns around. I think you simply have to accept that that’s part of life. (INT05)
Q2	• Well, I feel based off the traditional family model that the man is responsible for the family, right? As a matter of fact, we still have that in us a little bit, uh… (INT21) • It’s just my duty. I’m the mother, and this is just how it has to be, right? You’re just there, whether a child is young or he is older and had an accident. It’s just the way it is. I can’t say much more. I just think of it as normal. (INT11) • Yes, sure [I will continue to support the care recipient]. Till death do us part [laughs]. He’ll stay home as long as we can manage it. That is out of the question. (INT01)
Q3	• [as a middle school teacher] I was used to solving problems and helping others out or to check that everything is in order. That’s the way I am… I could always practice in my job in a different way, couldn’t I? (INT02) • It probably depends a little bit on the personality. Maybe I’m the kind of person who tends to help. I also do some volunteer work and stuff like that. (INT06) • I have a helping nature. (INT13) • I like helping people. (INT22)
Q4	• For me, it is somehow easier if I do it alone. (INT02) • Maybe I am also a person who doesn’t accept help so easily, who prefers to do everything himself. (INT08) • [speaking of home care providers] It’s always an intrusion, someone who comes into your house. It’s hard to accept that these people come twice a day. It’s hard, it’s an intrusion into your life, it’s difficult. (INT20) • This is always an interference in the family routine. At home you like to be alone, right? Every stranger who comes in is a foreign body, right? At some point you don’t want it anymore, so you try to avoid it. (INT21)
Q5	You kind of slip into it, don’t you? It was actually clear from the beginning, we were trained accordingly here [rehabilitation clinic]. (INT07) • After rehabilitation, she [care recipient] lived with the family, so she was never really dependent on external help, on home care providers. I slipped into it and I took over. (INT09) • I kind of slid into it. After the accident, I never once thought that I would delegate it to someone else. (INT08)
Q6	• Where do you put a child? […] we’ve looked for options. There was nothing. No places where such a young person could be, without suddenly having to live with elderly people. It was simply not imaginable. Actually, there was no other solution. (INT18) • From her [care recipient] side, it was actually clear from the beginning [that I would take over caregiving]. Having home care providers or moving to an assisted living facility was never a topic for discussion in her family. The situation was like this and I took it over. (INT09) • It’s also expected, she [care recipient] expects that we help and above all that the daughters help. That’s how it is. (INT05) • This actually happened quite quickly. It was also expected because I’m a nurse by training. For this reason it became clear relatively quickly, especially for my husband [care recipient], because he had difficulties in the beginning to delegate personal care to external people. (INT08)
Q7	• I only cried there because with each sentence I understood more and more that he [care recipient] would never walk again. I was just crying and crying. I couldn’t talk anymore and I couldn’t think. I didn’t want to listen to this information. (INT18) • We first had to find out what actually happened, what is still possible and what you can do. But you are in the dark and you search in the dark. At the beginning you experience like a crash. […] And you celebrate every small progress, like when she [care recipient] could move a finger just a little bit. Of course, you then hoped that she would improve even more. Unfortunately it didn’t happen [laughs]. (INT21) • At the beginning you still have the feeling that the situation will change, or that a surgery can fix it […] This was actually the worst time. […] Of course there was the hope that it would improve, recover. You somehow have some slightly abstruse ideas, that the lesion is temporary, or that it is something else. (INT04) • First of all, it takes a couple of weeks before you even realize what he [care recipient] has, until you realize, “Ah, that’s permanent.” It takes six months to have a final diagnosis and you realize: “Ah, there is no recovery, that’s permanent.” Just the first six months when you realize: “Jesus, it’s not improving.” (INT13)
Q8	• We haven’t felt very supported in our search for answers about tetraplegia and its consequences. What does it mean to be tetraplegic? What chances does a fifteen-year-old with such a diagnosis have for the future? We had to find that out all by ourselves. […] I think there should be something like a travel guide for newly injured people and their families because it is a journey, and a long one. (INT18) • What we’ve been missing is a little bit of education on the whole thing. We live in the countryside and there is a lack of support and education, and even doctors are a bit overwhelmed with the special problems of a wheelchair user. (INT01) • You have to search for information on your own on many topics. Today you can also read a lot of things on the internet and otherwise you just have to ask directly. (INT06)
Q9	• It’s all about the wheelchair user. At the beginning, family members receive some attention, but as soon as the situation is a bit more stable, nobody checks anymore. (INT13) • Everything that happened around my husband [care recipient], that was great. […] But I was not told clearly enough that it is important to learn to set limits from the beginning. I think that this would have helped a lot, there wouldn’t have been so many problems afterwards. (INT08)
Q10	• I used to have jobs for which I had to leave early in the morning. But it just didn’t work out, it was too chaotic. (INT13) • I retired earlier and we always said that when she would also retire, we would have the time for traveling. We always discussed about traveling in Scandinavia […] And of course it was a huge change afterwards [after the accident]. (INT07) • I actually gave up a good job because my willingness or desire to reduce my working time to 80% to be able to do more at home and be at home more wasn’t appreciated. So, in that sense you need to be ready to take a step backwards professionally. (INT21)
Q11	• Before knowing her, I used to work 100%. Then I got to know her, I moved, and I explicitly looked for a job which would allow me to stay at home in the morning for assisting her and go to work in the afternoon. (INT09)
Q12	• The day is structured this way: the morning is dedicated to grooming, in the afternoon I work and in the evening I do whatever else is needed. (INT09) • He [care recipient] needs this stable structure: get up, have breakfast, plan the day, and then around at eight o’clock I go to work. (INT13) • Monday and Tuesday I have to cook for him [care recipient] too. He works until 2:30 pm. Then he comes home, I give him something to eat and help him go to bed to relieve the skin because of pressure injuries. At 5 pm I help him get up and then I usually spend the evening with him […] And then between ten and eleven, I put him back to bed. (INT11) • I work 100%. I get up at 5 or 5:30am and I help my wife change position, then I leave. […] At about 6:30am the home care providers arrive […]. We have lunch at my mother-in-law’s place […]. After lunch, we go back home and I help her go to the toilet and transfer to the bed and I go to work. I come home at 5 pm or 6 pm and from this moment on, I do most of the work. I cook, then we go on the sofa, watch TV and then at 10 or 11 pm we go to bed. […] During the night, usually between 1am and 3am, we wake up because she needs to change position. (INT12)
Q13	• There was nothing. No places where such a young person could be, without suddenly having to live with elderly people. (INT18)
Q14	• I do have worry. I just retired, I’m turning 65 and then sometimes you think about the future, whether he’ll still be able to do it by himself, for instance in 20 years. You think about it, don’t you? (INT03) • The worry is there. I ask myself: what if something happens to me? Who’s looking after him? That’s quite a responsibility. Yeah, I’m starting to already think about it. (INT14)
Q15	• Last year I wished to go orienteering in Sweden. We then searched for a place where my wife could stay for three weeks and we found holiday room in a nursing home […] The drawback is that she now ended up here [rehabilitation clinic] with a pressure injury. (INT07)
Q16	• We had to argue with the insurance companies for a long time to get our rights. (INT08) • Once we asked the disability insurance to have a new shower wheelchair because the one we were given didn’t work. In the end, they paid for a new one, but you always have to insist. (INT14) • I was also a little disappointed by the disability insurance. […] it’s very bureaucratic […]. You might get an offer with conditions that are impossible to fulfill and then it is sometimes a little complicated until you find the right person in the right place, who will also understand that this is not possible and that you have to change something. (INT06) • Activating the payers and insurances, it was a huge effort […] Writing emails and emails and filling out forms […] The whole carousel goes round and round and I’m really exhausted. (INT18)
Q17	• I used the psychological services. That was after the shock of the accident. After the shock, you finally can breathe and think, what’s up now? And what I discovered, was that the insurance would not pay. But if I would have asked for psychological support immediately after the accident, the insurance would have paid. (INT08) • [speaking of psychological support] I couldn’t go forever because that’s limited. The health insurance doesn’t pay that much and then you needed a diagnosis to be able to have more sessions. (INT13)
Q18	• The disability insurance always questions everything: “Is this needed?” and so on. “Of course it’s needed, he [care recipient] is tetraplegic C4/C5”. You have to prove everything again and again, justify everything. My husband has developed a hate. He went from frustration to anger and today it is pure hate. He says: “If I could, I would put a bomb at the disability insurance.” He can’t stand this anymore and he’s a strong person. (INT18)
Q19	• It takes two to three years until they pay, when it is clear enough that you need it. (INT17) • The finances were not yet in balance, it took three years for the insurance companies to pay, three years. (INT13) • We needed a lot of money. Of course, the disability insurance contributed, but we also had to invest money for adapting the house. Or we often had to pre-finance the whole thing and wait a while for the reimbursement from the disability insurance. […] In addition, I gave up my job and this was an income loss and then we started to use our savings and, to a certain extent, also our retirement savings. Last year my husband would have retired but for this reason he continues to work. (INT18) • We’re okay, but I’m not travelling around the world or planning holidays, this doesn’t exist for me. My son [care recipient] has a small savings account thanks to his grandmother, but when this money is finished, it’s over. Then we have to see how things will go on. Because with my salary I cannot support two people. (INT14) • Unfortunately, we are now in the situation in which we nearly used all our savings. And we have to find a solution. It can’t go on like this. I have to earn more as soon as possible. There is a certain pressure. (INT21)
Q20	• Many people cannot imagine that you have to give up your own life. (INT01) • It has its drawbacks too, right? It all depends on me, of course. That’s a bit the thing, but that’s how it is. (INT09)
Q21	• I was a high school teacher and at some point I would have liked to change and do a training for working as a diplomat. It really interested me, I asked for the needed documents and then I had to tell myself that with a wife who is a wheelchair user I couldn’t do it. Move around the world and have a job somewhere, it’s just not very… not very realistic. (INT04) • I would have had wishes professionally, wishes which I could not fulfil and today I have an age for which it is too late, but that doesn’t matter, it’s all right. (INT18) • I could have actually done many exciting things in my career, but after the accident I kind of lost the freedom to take on all jobs. From that moment on, I made all my decisions by taking into consideration my partner or how we could do it together. (INT21)
Q22	• You are really limited in your life, from private perspective, for fun or pleasure, flexibility, spontaneity. (INT14) • Well, I can’t do certain things as spontaneously as others would. (INT21) • […] or just generally spontaneous decisions. If someone calls us and asks, “What about doing this today? Or we are nearby, would you like to join?” It doesn’t work because she drank and now she has to empty her bladder and so on. That’s a little bit what for sure gets lost. (INT09) • I’m a bit tied down. I can’t just decide on my own that tomorrow I’ll leave for a few days or so. (INT04) • I postpone all my wishes [laughs], for instance go out with friends. Or I love to travel, I like to get in touch with people and so on. But now I avoid almost everything. (INT22)
Q23	• As the mother I knew that I would not take over personal care because when you are 15, that is the age when you start to become independent and you no longer walk around naked in front of your mom. (INT18) • It was our idea from the beginning that she [care recipient] would dress herself and I won’t become a nurse. We are aware that it’s not ideal when the partner becomes the nurse. (INT21)
Q24	• At the beginning, we had no home care providers. I was doing everything alone. Then, at some point, I realized that this was not good for us. I was able to convince him to have someone helping at least twice a week. (INT08) • We don’t need to do so much grocery shopping anymore because we organized a meal delivery service for lunch. We made this decision because she forgot many times the pan on the stove [laughs] and then it was a little dangerous. (INT05)
Q25	• The things that give me pleasure have changed since the accident. I say that’s good for me, that’s not good for me anymore. Because you have less free time when you look after someone, so you have to make the best out of this time. (INT13) • We are both aware that we can pursue our own hobbies. But it’s always a tightrope walk, because it’s always a matter of weighing up: “Can I do that now?” But I don’t feel limited in what I’d like to do. (INT21) • I now have only 10% for my freedom, only 10%. Before I could go when I wanted. (INT22)
Q26	• I’m not flexible at all anymore. I can’t say last minute, “Today we’ll go shopping in [city] or we’ll go hiking.” That’s not possible and if it is possible, it involves an effort and then I think: “The heck with it, just stay at home.” I’ll do it, only if this is really important to me. (INT08)
Q27	• […] my partner cannot imagine spending a week or two with strangers […] But being frank, it would be for me certainly a relief if she would go away for a week [respite options for caregivers]. (INT09) • I would like my husband to go on holiday alone, but I haven’t managed to convince him yet. He doesn’t want to do it. (INT08)

**Fig. 1:**
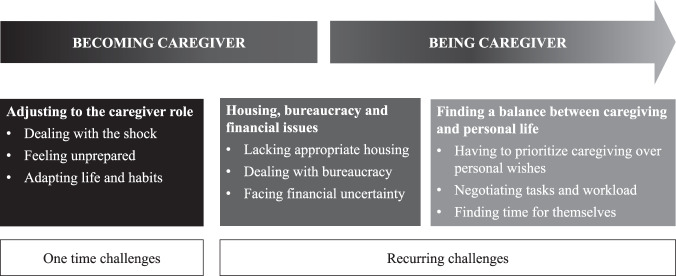
Challenges over time. Overview of the findings.

Despite the different ways in which the participants described the beginning of caregiving, they seemed to have experienced a number of similar challenges. The first phase was characterized by specific challenges that seemed to mirror how the caregiving started: unexpected and disruptive. Dealing with these challenges allowed the family member to embed the caregiver role into their life. The second phase was marked by recurrent challenges and subsequent adjustments. In the following, we provide an in-depth exploration of the challenges that characterize the lifelong process of becoming and being a caregiver (Fig. 1).

### Challenges related to adjusting to the role as caregiver

#### Dealing with the shock

In situations in which the care recipient and caregiver already had a relationship before SCI onset (e.g., parent–child, partners), the analysis highlighted that the caregiver had to deal with shock and realize that the disability was permanent. The participants reported that during the acute rehabilitation phase they had to understand what happened, manage their expectations for recovery, and start coming to terms with the situation (Q7).

#### Feeling unprepared

The participants reported a lack of information, training, and guidance (Q8). When looking back, some interviewees stated that the focus was always on the person with SCI and resented a lack of support in preparing them for a “lifetime job” as a caregiver (Q9).

#### Adapting life and habits

Becoming a caregiver required adaptations in many domains: professional life (e.g., reducing working time), social life (e.g., less time for friends and hobbies), and family life (e.g., moving because the house could not be adapted) (Q10). Even in the cases of caregivers who took on the task years after SCI onset, there was a need to adapt their lives to the new situation (Q11). Adapting their lives and habits gave rise to new routines, which were often fixed and based around the needs of the care recipient (e.g., self-management activities such as emptying the bladder or laying down to prevent pressure injuries) (Q12).

### Challenges related to housing, bureaucracy, and financial issues

#### Lacking appropriate housing

Housing for people with SCI are lacking, in particular for young people, for whom neither nursing homes nor assisted living facilities for individuals with learning disabilities are adequate solutions (Q13). Moreover, caregivers who had started to feel the consequences of ageing wondered about who would take on caregiving when they are no longer able to provide the needed care. This worry especially affected caregivers whose care recipient was not accustomed to receiving assistance from homecare providers (Q14). In some cases, this worry was reinforced by bad experiences with nursing care facilities (Q15).

#### Dealing with bureaucracy

Several participants mentioned the challenges of dealing with the bureaucracy of insurance providers. They mostly complained about the complexity of the reimbursement system (Q16) or the strict rules (e.g., reimbursement for psychological support) (Q17). Some interviewees also stressed how mentally exhausting the process of applying for reimbursement or allowances could be (Q18).

#### Facing financial uncertainty

The participants reported that the often very long procedures to define disability benefits left families in limbo and facing financial uncertainty. The situation could be more or less difficult, depending on their financial situation prior to SCI onset and the caregiver’s work situation. Among the participants facing financial difficulty, some decided to continue working even after reaching official retirement age. Among those who were in a comfortable situation, some expressed worry for the future (Q19).

### Challenges related to finding a balance between caregiving and personal life

#### Having to prioritize caregiving over personal wishes

Some participants acknowledged that caregiving required sacrifice in terms of personal aspirations and freedom (Q20). Some interviewees stated that they had to give up vocational retraining or new professional experiences because these were not compatible with their caregiver role (Q21). Several others reported having to refuse invitations or give up activities when these did not fit with their caregiving schedule. Many caregivers acknowledged that caregiving affected their ability to make spontaneous plans (Q22).

#### Negotiating tasks and workload

Some caregivers described how they negotiated their workload and tasks (Q23). For example, the fear of mixing the roles of partner and caregiver led to a clear division of tasks: personal care (e.g., bowel management) was delegated to homecare providers, while the partner took on assistance (e.g., mobility). The analysis highlighted that this negotiation is an iterative process and can be triggered by several factors; for example, caregivers might realize that the situation is not sustainable anymore because of ageing, other commitments, or unclear role boundaries (e.g., being caregiver, life partner, and working partner), or care recipients might develop new needs that require new arrangements (Q24).

#### Finding time for themselves

Caregiving was often perceived as a full-time job, and a major challenge for caregivers was to find time for themselves (Q25). With good organization, it was possible to create free time, but such organization could be demanding. Some caregivers stressed that an activity had to be worthwhile; otherwise, the planning effort exceeded the pleasure (Q26). Furthermore, in many cases this would require the care recipient to accept external support, which was often undesired (Q27).

## Discussion

This study found that SCI caregiving most often begins unexpectedly and is characterized by two phases. The first phase is relatively short and central to becoming a caregiver, and it is marked by challenges related to adjusting to the role of caregiver and embedding this in their lives. The second phase, which is lifelong, is characterized by a number of recurrent challenges related to finding a balance between caregiving and personal life. The challenges related to housing, bureaucracy, and financial issues take place in both phases. Caregivers have to deal with these challenges to stay apprised of life changes (e.g., retirement) and newly emerging needs (e.g., how to deal with ageing in SCI).

These finding have two major implications. First, by describing how family members become caregivers, a time point in which vulnerable caregivers can be identified is suggested. Second, by describing the challenges and their timing, inputs for developing tailored programs and assessing the adequacy of the services available are provided.

In relation to the first point, the “birth” of a caregiver offers the earliest opportunity to identify people in need of support. Indeed, as past research has documented, becoming a caregiver is a turning point in one’s life trajectory [22], and for relatives, it seems to be less a matter of choice than a matter of responsibility and reciprocity [23, 24]. Considering that feelings of control in caregiving relate to caregivers’ wellbeing and that the decision to become a caregiver is an indicator of control [25], caregivers who choose to take on caregiving might experience higher levels of control than caregivers who “slipped into it”. In addition, research has shown that less control in caregiving is associated with a lower household income, a lower subjective social position, and a higher objective caregiver burden [25]. Thus, it might be important to assess these factors to identify vulnerable caregivers in a timely manner.

The moment a person becomes a caregiver is a turning point. However, our findings indicate that being a caregiver is more a process than a state. Consequently, caregivers have to find resources throughout their lives to assist the care recipient without jeopardizing their own well-being. In line with earlier research [3, 26, 27], we believe that routine need-and-outcome assessments may be useful. In practice, rehabilitation clinics could offer a “caregiver checkup” in tandem with the checkup offered to the people with SCI. This is in accordance with Chan [28], who stressed the importance of considering a caregiver and care recipient as a single unit to promote understanding and preparedness post discharge.

During the “caregiver checkup”, caregivers could be screened with regard to the challenges they may be facing and the care recipient’s health status and functional independence, as these are associated with caregiver outcomes [29, 30]. Such support might not only benefit caregivers, it could also positively influence the care recipient and the relationship between the two [13, 31]. Furthermore, positive caregiver outcomes would ensure the caregiving is sustainable, thereby contributing greatly to the healthcare system.

The findings of this study might also provide an opportunity to check if the services available support caregivers in addressing the challenges they encounter over time. While adjusting to their new role, caregivers often feel unprepared and struggle with shock. Therefore, the effectiveness of educational programs in increasing caregivers’ confidence and competence in providing safe and effective care could be evaluated, as could the services addressing distress [32, 33]. Similarly, the adequacy of services to support caregivers in dealing with challenges linked to housing, bureaucracy, financial issues, and balancing caregiving and personal life needs to be assessed. In Switzerland, several services (e.g., homecare providers, respite care) are available, but their use is limited and dependent on the characteristics of both the caregiver and the care recipient [2, 34]. In about half of all cases, there seems to be no need of these services; however, sometimes costs, limited flexibility and care receiver’s rejection of external assistance are barriers to using these services [2].

Furthermore, our findings suggest that the timing of existing services should be considered. Indeed, a limitation is that most of these services, including caregiver education, are offered during or shortly after discharge from acute care or rehabilitation [35]. For educational programs this is problematic, as receiving too much information at once or at the wrong time might not be helpful [36] (p.153). Hence, we endorse the suggestion of Graf and colleagues [26] to offer support at different points in time. Based on the results of a “caregiver checkup”, rehabilitation clinics could identify caregivers in need and direct them to the appropriate services or provide them with tailored information. Linking caregivers to resources would help them find the appropriate support when it was most needed. In this regard, the introduction of case management programs could also help; in a recent study in Switzerland nearly half of the respondents reported that their need for case management was at best partially fulfilled [37].

This study has some methodological limitations. First, it relied on the memories of participants, who were reporting their (often very long) caregiving experiences. Prospective longitudinal studies could further explore the specific challenges of caregiving directly after first rehabilitation; however, the fact that saturation was reached supports the existence of several shared challenges. Second, the composition of our sample did not allow for performing comparisons among groups (e.g., caregivers with different relationships with the care recipient, male vs female caregivers). It is nonetheless important to notice that in Switzerland caregivers of people with SCI are mostly life partners and female [2], as in our sample. Finally, some of the challenges presented in this article might be specific to the Swiss context.

## Conclusion

The findings of this study suggest that SCI caregivers go through two phases and that their lives are characterized by continuous adjustment to the ever-evolving role of caregiver. Furthermore, describing how family members become caregivers and the challenges they face over time might help improve the societal response to SCI by developing tailored interventions aimed at equipping caregivers with the necessary knowledge and skills. This will not only benefit caregivers, it is also likely to have a positive impact on the care recipient and the relationship between the two. Caring for caregivers is a way of giving back to these family members who support the healthcare system with their often invisible, but extremely valuable, work.

### Data archiving

The transcripts of the interviews analyzed in the current study are available from the corresponding author on request. Transcripts will be provided in the original language (mostly German).

## Supplementary information

Consolidated criteria for reporting qualitative studies (COREQ 32-item checklist)

## References

[1] Smith EM, Boucher N, Miller WC (2016). Caregiving services in spinal cord injury: a systematic review of the literature. Spinal Cord.

[2] Gemperli A, Rubinelli S, Zanini C, Huang J, Brach M, Pacheco et al. Family caregivers of persons with spinal cord injury. J of Rehabilit Med. (in press). 10.2340/16501977-2762 33119123

[3] Post M, Bloemen J, De Witte L (2005). Burden of support for partner of persons with spinal cord injuries. Spinal Cord.

[4] Houldin AD (2007). A qualitative study of caregivers’ experiences with newly diagnosed advanced colorectal cancer. Oncol Nurs forum.

[5] Grimmer K, Moss J, Falco J (2004). Becoming a carer for an elderly person after discharge from an acute hospital admission. The Internet J of Allied Health Sci and Prac.

[6] Silva-Smith AL (2007). Restructuring life: preparing for and beginning a new caregiving role. J Fam Nurs.

[7] Schulz R, Sherwood PR (2008). Physical and mental health effects of family caregiving. Am J Nurs.

[8] Brewin A (2004). The quality of life of carers of patients with severe lung disease. Br J Nurs.

[9] Lynch J, Cahalan R (2017). The impact of spinal cord injury on the quality of life of primary family caregivers: a literature review. Spinal Cord.

[10] Baker A, Barker S, Sampson A, Martin C (2017). Caregiver outcomes and interventions: a systematic scoping review of the traumatic brain injury and spinal cord injury literature. Clin Rehabil.

[11] Savic G, DeVivo M, Frankel H, Jamous M, Soni B, Charlifue S (2017). Long-term survival after traumatic spinal cord injury: a 70-year British study. Spinal Cord.

[12] International perspectives on spinal cord injury. World Health Organization & The International Spinal Cord Injury Society 2013.

[13] Tough H, Brinkhof MW, Siegrist J, Fekete C (2017). Subjective caregiver burden and caregiver satisfaction: the role of partner relationship quality and reciprocity. Arch Phys Med Rehabil.

[14] Fekete C, Tough H, Siegrist J, Brinkhof MW (2017). Health impact of objective burden, subjective burden and positive aspects of caregiving: an observational study among caregivers in Switzerland. BMJ Open.

[15] Post MW, Brinkhof MW, von Elm E, Boldt C, Brach M, Fekete C, et al. Design of the Swiss spinal cord injury cohort study. Am J Phys Med Rehabil. 2011;90:5–16. 10.1097/PHM.0b013e318230fd41 21975676

[16] Brinkhof MW, Fekete C, Chamberlain JD, Post MW, Gemperli A (2016). Swiss national community survey on functioning after spinal cord injury: Protocol, characteristics of participants and determinants of non-response. J Rehabil Med.

[17] Lai DWL (2012). Effect of financial costs on caregiving burden of family caregivers of older adults. SAGE Open.

[18] Hu X, Dolansky MA, Zhang F, Qu M (2016). Factors associated with the caregiver burden among family caregivers of patients with heart failure in southwest China. Nurs Health Sci.

[19] Saunders MM (2008). Factors associated with caregiver burden in heart failure family caregivers. West J Nurs Res.

[20] Adelman RD, Tmanova LL, Delgado D, Dion S, Lachs MS (2014). Caregiver burden: a clinical review. JAMA.

[21] Braun V, Clarke V (2006). Using thematic analysis in psychology. Qualitative Res Psychol.

[22] Mandelbaum DG (1973). The study of life history: gandhi. Curr Anthropol.

[23] Pertl MM, Sooknarine-Rajpatty A, Brennan S, Robertson IH, Lawlor BA (2019). Caregiver choice and caregiver outcomes: a longitudinal study of Irish spousal dementia caregivers. Front Psychol.

[24] Schulz R, Beach SR, Cook TB, Martire LM, Tomlinson JM, Monin JK (2012). Predictors and consequences of perceived lack of choice in becoming an informal caregiver. Aging Ment Health.

[25] Fekete C, Tough H, Brinkhof MWG, Siegrist J (2019). Does well-being suffer when control in productive activities is low? A dyadic longitudinal analysis in the disability setting. J Psychosom Res.

[26] Graf R, LeLaurin J, Schmitzberger M, Freytes IM, Orozco T, Dang S (2017). The stroke caregiving trajectory in relation to caregiver depressive symptoms, burden, and intervention outcomes. Top Stroke Rehabilitation.

[27] Stucki G, Bickenbach J (2017). The implementation challenge and the learning health system for SCI initiative. Am J Phys Med Rehabil.

[28] Chan RCK (2000). How does spinal cord injury affect marital relationship? A story from both sides of the couple. Disabil rehabilitation.

[29] Springate BA, Tremont G (2014). Dimensions of caregiver burden in dementia: impact of demographic, mood, and care recipient variables. Am J Geriatr Psychiatry.

[30] Lu N, Liu J, Lou VWQ (2016). Exploring the reciprocal relationship between caregiver burden and the functional health of frail older adults in China: a cross-lag analysis. Geriatr Nurs.

[31] Bakas T, Clark PC, Kelly-Hayes M, King RB, Lutz BJ, Miller EL (2014). Evidence for stroke family caregiver and dyad interventions: a statement for healthcare professionals from the American Heart Association and American Stroke Association. Stroke.

[32] Reinhard S, Given B, Petlick N, Bemis A. Supporting family caregivers in providing care. In: Hughes R, editor. patient safety and quality: an evidence-based handbook for nurses. Rockville: Agency for Healthcare Research and Quality 2008. 21328765

[33] Chien LY, Chu H, Guo JL, Liao YM, Chang LI, Chen CH (2011). Caregiver support groups in patients with dementia: a meta-analysis. Int J Geriatr psychiatry.

[34] Huang J, Pacheco Barzallo D, Rubinelli S, Münzel N, Brach M, Gemperli A. What influences the use of professional home care for individuals with spinal cord injury? A cross-sectional study on family caregivers. Spinal Cord. 2019;57:924–32. 10.1038/s41393-019-0296-y PMC6892416 31127196

[35] Visser-Meily A, van Heugten C, Post M, Schepers V, Lindeman E (2005). Intervention studies for caregivers of stroke survivors: a critical review. Patient Educ counseling.

[36] Coon DW, Gallagher-Thompson D, Thompson LW (2003). Innovative interventions to reduce dementia caregiver distress: a clinical guide.

[37] Trezzini B, Brach M, Post M, Gemperli A (2019). Prevalence of and factors associated with expressed and unmet service needs reported by persons with spinal cord injury living in the community. Spinal Cord.

