# Supplementary material for: The challenges characterizing the lived experience of caregiving. A qualitative study in the field of spinal cord injury
Source: Spinal Cord. 2021 Mar 19;59(5):493–503. doi: 10.1038/s41393-021-00618-4 (PMC8110474; doi:10.1038/s41393-021-00618-4)
Supplement: Supplementary file 1 — Consolidated criteria for reporting qualitative studies (COREQ 32-item checklist) [file 41393_2021_618_MOESM1_ESM.pdf]

Zanini C, Amann J, Brach M, Gemperli A, Rubinelli S. The challenges characterizing the lived experience of caregiving. A qualitative study in the field of spinal cord injury. *Spinal Cord*. 2021.

Supplementary file 1: Consolidated criteria for reporting qualitative studies (COREQ 32-item checklist)

For reason of space, not all items are reported in the manuscript. In the table below, the authors inserted complementary information.

**No. Item**	**Guide questions/description**	**Section in the manuscript**
**Domain 1: Research team and reflexivity**		
Personal Characteristics		
1. Interviewer/facilitator	Which author/s conducted the interviews?	Methods
2. Credentials	What were the researcher's credentials? E.g. PhD, MD	Not reported in the manuscript.<br>*CZ and JA have a PhD in Health Sciences*
3. Occupation	What was their occupation at the time of the study?	Not reported in the manuscript.<br>*Senior researchers*
4. Gender	Was the researcher male or female?	Not reported.<br>*Female*
5. Experience and training	What experience or training did the researcher have?	Not reported in the manuscript.<br>*CZ and JA were trained during their studies as well as during their 3-year graduate program. They conducted several qualitative studies both for their PhD projects and in relation to other projects. NL had a MA in Health Sciences and experience in recruitment and data collection (interviews and focus groups) thanks to an internship in our research institute.*
Relationship with participants		
6. Relationship established	Was a relationship established prior to study commencement?	Not reported in the manuscript.<br>*The interviewers did not know the interviewees prior to the study*.
7. Participant knowledge of the interviewer	What did the participants know about the researcher? e.g.	Not reported in the manuscript.<br>*The participants knew the credentials of the researcher*.

	personal goals, reasons for doing the research	
8. Interviewer characteristics	What characteristics were reported about the inter viewer/facilitator? e.g. Bias, assumptions, reasons and interests in the research topic	Not reported in the manuscript.<br>*The interviewers kept a diary (see below).*
**Domain 2: study design**		
Theoretical framework		
9. Methodological orientation and Theory	What methodological orientation was stated to underpin the study? e.g. grounded theory, discourse analysis, ethnography, phenomenology, content analysis	Methods
Participant selection		
10. Sampling	How were participants selected? e.g. purposive, convenience, consecutive, snowball	Methods
11. Method of approach	How were participants approached? e.g. face-to-face, telephone, mail, email	Methods
12. Sample size	How many participants were in the study?	Results
13. Non-participation	How many people refused to participate or dropped out? Reasons?	Not reported in the manuscript.<br>*Three potential participants explicitly refused to participate because of lack of time, although they indicated in the survey their availability. No participant dropped out.*

Setting		
14. Setting of data collection	Where was the data collected? e.g. home, clinic, workplace	Methods
15. Presence of non-participants	Was anyone else present besides the participants and researchers?	Not reported in the manuscript.<br>*No one was present except the interviewer and the interviewee.*
16. Description of sample	What are the important characteristics of the sample? e.g. demographic data, date	Results
Data collection		
17. Interview guide	Were questions, prompts, guides provided by the authors? Was it pilot tested?	*Methods*<br>*Complementary information: The interview guide was developed by CZ and revised in collaboration with the research team. The questions were developed with the aim to explore the lived experience of SCI caregivers. Knowing from the literature that caregiving often disrupts the life of the caregiver and that caregivers reported, among others, high level of burden, physical symptoms, and reduced life satisfaction, we included questions that covered these aspects. In addition, and in line with other scientific literature, we asked questions to explore the positive aspects of caregiving, which aimed to uncover the resources and strategies that caregivers put into place.*<br><br>*The questions would guide the conversation but there was the opportunity to follow up on issues raised by the participants. NL conducted the three pilot interviews to test the interview guide. No major changes were made in the interview guide, but the wording was improved to avoid misunderstandings. We therefore included the interviews in the analysis.*
18. Repeat interviews	Were repeat interviews carried	*No*

	out? If yes, how many?	
19. Audio/visual recording	Did the research use audio or visual recording to collect the data?	Methods
20. Field notes	Were field notes made during and/or after the interview?	Not reported in the manuscript.<br>*The interviewers kept a diary. After every interview, notes were taken on the following aspects: setting, feelings during the interview and first impressions. They might also take notes of initial analysis thoughts, interpretations, and questions. The interviewers also regularly exchanged between them on these topics.*
21. Duration	What was the duration of the interviews?	Methods
22. Data saturation	Was data saturation discussed?	Methods / Discussion
23. Transcripts returned	Were transcripts returned to participants for comment and/or correction?	No
**Domain 3: analysis and findings**		
Data analysis		
24. Number of data coders	How many data coders coded the data?	Methods<br>*Complementary information: The first author (CZ) conducted the first deductive coding of six interviews and JA and NL of each three interviews. CZ sat then with JA and with NL to compare the coding and solve disagreement. The same procedure was followed for the inductive coding. In this case, the three researchers met several times to develop a first bunch of shared codes. CZ continued then the coding process alone and regularly met with NL and JA to present the findings (always supported by quotes from the interviews) and gather inputs to work on their consistency.*
25. Description of the	Did authors provide a	No

coding tree	description of the coding tree?	
26. Derivation of themes	Were themes identified in advance or derived from the data?	Methods
27. Software	What software, if applicable, was used to manage the data?	Methods
28. Participant checking	Did participants provide feedback on the findings?	No
Reporting		
29. Quotations presented	Were participant quotations presented to illustrate the themes/findings? Was each quotation identified? e.g. participant number	Results
30. Data and findings consistent	Was there consistency between the data presented and the findings?	Results
31. Clarity of major themes	Were major themes clearly presented in the findings?	Results
32. Clarity of minor themes	Is there a description of diverse cases or discussion of minor themes?	Results

Developed from:

Tong A, Sainsbury P, Craig J. Consolidated criteria for reporting qualitative research (COREQ): a 32-item checklist for interviews and focus groups. *International Journal for Quality in Health Care*. 2007. Volume 19, Number 6: pp. 349 – 357
